# Intelligent Identification of Coal Crack in CT Images Based on Deep Learning

**DOI:** 10.1155/2022/7092436

**Published:** 2022-09-23

**Authors:** Jinxia Yu, Chengyi Wu, Yingying Li, Yimin Zhang

**Affiliations:** ^1^School of Computer Science and Technology, Henan Polytechnic University, Jiaozuo 454000, China; ^2^State Key Laboratory Cultivation Base for Gas Geology and Gas Control, Henan Polytechnic University, Jiaozuo 454000, China; ^3^School of Computer and Information Engineering, Luoyang Institute of Science and Technology, Luoyang 471023, China

## Abstract

Automatic segmentation of coal crack in CT images is of great significance for the establishment of digital cores. In addition, segmentation in this field remains challenging due to some properties of coal crack CT images: high noise, small targets, unbalanced positive and negative samples, and complex, diverse backgrounds. In this paper, a segmentation method of coal crack CT images is proposed and a dataset of coal crack CT images is established. Based on the semantic segmentation model DeepLabV3+ of deep learning, the OS of the backbone has been modified to 8, and the ASPP module rate has also been modified. A new loss function is defined by combining CE loss and Dice loss. This deep learning method avoids the problem of manually setting thresholds in traditional threshold segmentation and can automatically and intelligently extract cracks. Besides, the proposed model has 0.1%, 1.2%, 2.9%, and 0.5% increase in Acc, mAcc, MioU, and FWIoU compared with other techniques and has 0.1%, 0.8%, 2%, and 0.4% increase compared with the original DeepLabV3+ on the dataset of coal CT images. The obtained results denote that the proposed segmentation method outperforms existing crack detection techniques and have practical application value in safety engineering.

## 1. Introduction

Coal is an important energy source for human society. The phenomenon of deformation and damage of coal and rock mass under load is common, which has a huge impact on the safety of mining engineering. The research on digital core technology based on industrial CT scanning technology is of great significance for the mining safety, and its basis is the high-precision segmentation of cracks in industrial CT scanning images. As the key technology of digital core, 3D reconstruction needs high-precision segmentation results to reflect the original topology of cracks. However, artificial segmentation of coal crack CT images undoubtedly takes a lot of time and energy. And, most of the existing auxiliary software is based on traditional threshold segmentation methods which are still impossible to get rid of the interference of noise even working with some image preprocessing methods. Therefore, intelligent and automated segmentation of coal crack CT images is particularly important.

Digital images contain a lot of important information, which can be extracted in different ways in different fields. For example, it can be used in the field of encryption technology [1, 2], information security [3–6], in the field of industrial engineering [7], in the field of agriculture [8], and so on. Digital image processing technology includes many categories [9, 10], and image segmentation is one of them. Noise is one of the difficulties in the segmentation of coal crack images. In order to reduce the noise and enhance images, methods that were applied in the segmentation of crack include morphological filter [11], wavelet transforms [12, 13], anisotropic diffusion filter [14], and so on. However, many noises cannot be fundamentally removed by the traditional methods. Machine learning algorithms can achieve automatic crack detection and segmentation to a certain extent including structured forests [15, 16], minimal path selection [17], support vector machine [18], etc. Nevertheless, most features in machine learning need to be identified by experts and hand-coded. Deep learning models have powerful learning ability which can automatically complete the tasks of classification, detection, and segmentation after training. Starting from FCN [19], many high-performance semantic segmentation models have emerged such as U-net [20], SegNet [21], and PSPNet [22]. These models are based on convolution operations, apply feature extraction networks as backbones, and incorporate multiscale semantic information to achieve pixel-by-pixel segmentation of images. Deep learning methods have been applied in different crack segmentation fields [23–25] nowadays.

In this work, we present an end-to-end coal crack CT image segmentation method based on the deep learning model DeepLabV3+ [26]. Compared with existing deep learning methods, postprocessing is unnecessary for our method. Besides, our method achieves better performance on some evaluation metrics. These advantages are meaningful for the subsequent 3D reconstruction work and the establishment of digital cores.

## 2. Related Work

**Figure 1:**
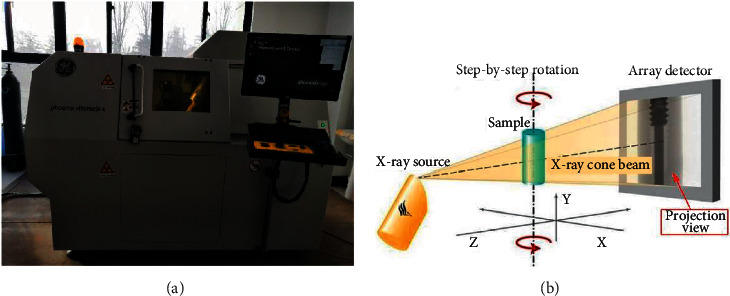
(a) High-precision industrial CT scanning equipment; (b) Schematic diagram of the scanning device.

**Figure 2:**
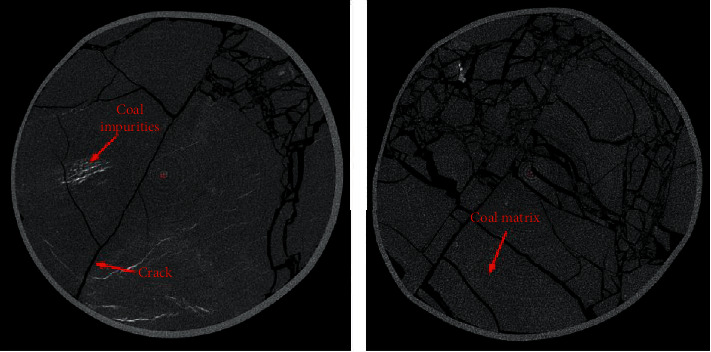
CT image samples of coal crack.

All data in this paper comes from the tomograms of high-precision industrial CT during the fracturing experiment of coal samples. The CT scanning equipment is from the Nation Key of Natural Gas Geology and Natural Gas Control of the Henan University of Technology Laboratory which is a phoenix v|tome|xm high-resolution industrial X-ray *μ*CT scanner [27]. The equipment diagram and CT imaging principle are shown in Figure 1. Images collected by this equipment have many noise points in the coal matrix, and different samples may have different colors. CT image samples of coal cracks are shown in Figure 2. The datasets used for training in this experiment are cut from CT images obtained by the aforementioned platform at different sizes. The diversity of the crack structure is fully considered in the interception process to adapt to the segmentation of different images.

The high performance of deep learning in computer vision was first demonstrated in classification tasks. Many CNN models can provide good classification accuracy such as Vgg [28], ResNet [29], Xception [30], and so on. Some of them are applied as feature extractors in segmentation models. FCN replaces the fully connected layer in the classification model with deconvolution to upsample the pooled feature map to its original size, pioneered semantic segmentation. The application of deep learning in crack detection can be roughly divided into three types, methods based on classification [31], object detection [32], and semantic segmentation [33, 34]. Xue et al. [35] modified the last few deconvolution modules of FCN to adapt to the needs of crack segmentation. However, this FCN-based method may not be able to guarantee the accuracy of segmentation and maintain the original topological structure of the crack when facing the crack of complex structures.

DeepLabV3+ is a high-performance semantic segmentation model derived from DeepLabV1, V2, and V3 [36–38]. In view of the adverse effect of excessive downsampling on segmentation accuracy, this model proposed to use atrous convolution to reduce downsampling and enlarge the receptive field simultaneously. This model also applied atrous spatial pyramid pooling to capture and fuse multi-scale semantic information which is beneficial to improving the accuracy of segmentation. Besides, Encoder-Decoder architecture is used to recover pixels of features better. DeepLabV3+ achieves new state-of-the-art performance on PASCAL VOC 2012 dataset. However, compared with the public semantic segmentation dataset, the crack image dataset has the characteristics of smaller targets and unbalanced positive and negative samples. So we have made some improvements to the original model for these characteristics. The coal crack CT image segmentation method that we proposed has the following contributions:

1. Given that there are no publicly available datasets for research in this field, we established a dataset of coal crack CT images for our research. All original pictures come from a professional coal sample fracturing experimental platform and all labels are made by hand marking.
2. We modified DeepLabV3+ to adapt to the need for coal crack CT images by adjusting the OS of the backbone, adjusting the encoder-decoder module, and changing the rates of the ASPP module. The modified model achieves better performance than the original model under some authoritative evaluation indicators commonly used in semantic segmentation: PA, MPA, MIoU, and FWIoU.
3. A new loss function is defined by combining the CE loss and Dice loss. While adding contour factors to the prediction, the curve fluctuation of the Dice function in the training is alleviated.

## 3. Methodologies

### 3.1. Dataset

**Figure 3:**
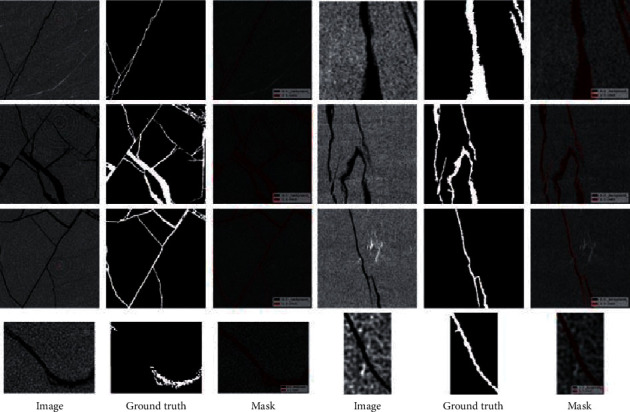
Some representative images in our datasets, their ground truth, and masks on original images. It shows that these pictures show that our dataset contains data of different sizes, different complexities, and different background color depths at the same time. Diversity allows the model trained on this dataset to adapt to most CT image environmental conditions.

Since there is no open-source dataset for CT segmentation images of coal crack, we established a coal crack dataset manually. All these images were taken from the original coal fracturing experimental images in different sizes and different length-width ratios. All data were captured in images acquired by high precision industrial CT introduced before. It consists of 437 RGB images and their segmentation labels, including different crack shapes, complexities, and different background colors. Some representative images and their annotations are shown in Figure 3. These samples can reflect the complexity of crack morphology, noise situation, and background differences in the dataset to a certain extent.

Data augmentation is a technique widely used in deep learning. In supervised learning, fine data annotation is a time-consuming and energy consuming work. Data augmentation can expand the dataset so that the parameters learned during model training are more reliable and can effectively avoid overfitting. So we enhanced the coal crack dataset to 5000 in different ways: rotation, flip and zoom. Angles of rotation were limited to −30 to 30 degrees, the flip direction is horizontal and the ratios of zoom were limited to 80% to 120%. Finally, after data augmentation, the training set contains 3500 images and the test set contains 1500 images.

### 3.2. Atrous Spatial Pyramid Pooling

Atrous convolution can be used to capture multiscale contextual information. The parameter can set different dilation rates of atrous convolution which can be regarded as the stride of the input signal we sample. The output of atrous convolution of a one-dimensional input signal with a filter of length is defined as follows:

(1) $$\begin{array}{c} y\left[i\right]=\overset{K}{\underset{k=1}{\sum }}x\left[i+r\cdot k\right]w\left[k\right]\text{.} \end{array}$$

**Figure 4:**
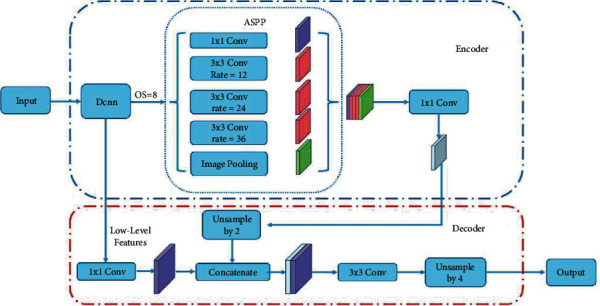
Modified DeepLabV3+ model structure. Compared with the original DeepLabV3+ architecture, the OS (output stride) was adjusted to 8, and rates of the ASPP module were adjusted to (1, 12, 24, 36). At the same time, the first upsampling rate is changed from 4 to 2 to restore image pixels to their original size.

Combined with spatial pyramid pooling, ASPP is applied as a Multiscale information fusion module. The structure which is applied in the DeepLabV3+ model achieved multi-scale information collection using four different rates of atrous convolutions (including image-level pooling). Different from the rates of (1, 6, 12, and 18) used in the original DeepLabV3+, more kinds of combinations of rates were tried using to make the feature extractor more suitable for crack segmentation. As the OS (Output Stride) of the backbone was adjusted to 8 to reduce downsampling, a larger receptive field is required. We tried to make the enlargement of the receptive field follow the size of the feature map output from the backbone. And, the experiment proved that rates of (1, 12, 24, 36) can achieve a better performance. A more intuitive situation about ASPP can be seen in Figure 4.

### 3.3. Encoder-Decoder

The encoder-decoder structure is widely applied in the field of computer vision. As for the semantic segmentation field, the encoder gains semantic information from images with feature maps reducing as a feature extraction module. DeepLabV3+ model uses DeepLabV3 as the encoder block with some effective improvement. The decoder is applied to reconstruct the segmentation result by restoring the pixel and size of the feature map, at the same time, keeping the details of the original image as much as possible. DeepLabV3+ proposed a simple decoder as shown in Figure 4 and obtained a good effect practically. The first upsampling rate was adjusted to 2 as the OS of the backbone was changed to 8.

### 3.4. Adjusted Xception as Backbone

Xception, as a high-performance convolution neural network is applied as the feature extractor of ordinary DeepLabV3+. This deep structure is developed based on the Inception model and based entirely on depthwise separable convolution. Unlike conventional convolution, in depthwise separable convolution, each feature map channel only needs to perform an operation with each channel of the convolution kernel. This kind of convolution can effectively reduce the number of parameters and computing costs, and by using this, Xception expanded the scale of the model and became state-of-the-art CNN architecture in classification tasks. The ordinary Xception has an OS = 32 so that it can adapt to the needs of classification tasks. But excessive pooling makes the feature maps too small so that the detailed information can be damaged. In order to get dense feature maps, the OS of 16 or 8 is desirable.

**Figure 5:**
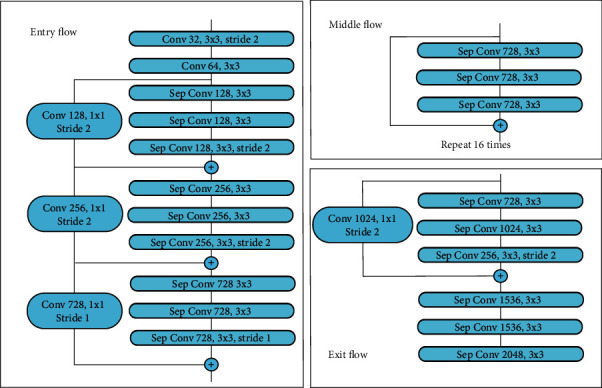
Modified Xception architecture. OS of the third block was adjusted to 1 to change the OS of the overall Xception to 8.

Different from the OS of 16 which performed better in natural scene datasets, crack images need a denser way to extract features because the targets of these images are tiny in most cases. For these small targets, downsampling has a particularly serious loss of accuracy. So the OS of 8 was used in this model, at the same time, the ASPP was adjusted to get a larger receptive field and the decoder also made corresponding adjustments. To achieve this goal, compared with the Xception structure in the DeepLabV3+ original text, we adjusted the stride of the third block of entry flow to 1, and correspondingly doubled the rate of the atrous convolution in the middle flow and the exit flow. The adjusted Xception structure is shown in Figure 5.

### 3.5. Loss Function

DeepLab series model apply the cross-entropy (CE) loss function which is widely applied in classification tasks to classify every single pixel. This loss function checks each pixel separately and compares the class prediction (the pixel vector in the depth direction) with the hot encoding target vector. The cross-entropy function can be formulated as follows:

(2) $$\begin{array}{c} L_{ce}=-\frac{1}{N}\underset{i}{\sum }\overset{M}{\underset{c=1}{\sum }}y_{ic}\log\left(p_{ic}\right), \end{array}$$

where *M* refers to the number of categories, *y*_*ic*_ refers to the sign function (0 or 1), and *p*_*ic*_ refers to the predicted probability that the observed sample *i* belongs to category *c*. Thus, we can consider that the pixels in the image are learned equally with the cross-entropy loss function, and this kind of equality does not apply to the situation where the sample is extremely uneven. In coal crack CT images, the number of pixels corresponding to the crack is much smaller than that of the background. Taking the dataset we established as an example, the proportion of crack pixels in the whole image is less than 5%. Dice Loss [39] was proposed in 2016, designed to deal with scenarios where positive and negative samples are strongly imbalanced in semantic segmentation. Different from distribution-based cross-entropy loss, the Dice function is based on region and is used to calculate the similarity between two images. The Dice coefficient and Dice loss function can be formulated as follows:

(3) $$\begin{array}{c} di\;ce=\frac{2\left|X\cap Y\right|}{\left|X\right|+\left|Y\right|}\text{,}\\ L_{di\;ce}=1-\frac{2\left|X\cap Y\right|}{\left|X\right|+\left|Y\right|}\text{,} \end{array}$$

where *X* and *Y* refer to two different samples, they are ground truth and predict mask in segmentation tasks. In a different way, the Dice coefficient and the loss function can be formulated as follows:

(4) $$\begin{array}{c} di\;ce=\frac{2TP}{2TP+FP+FN}\text{,}\\ L_{di\;ce}=1-\frac{2TP}{2TP+FP+FN}\text{.} \end{array}$$

 Where *FP*, *FN* refer to true positive, false positive, and false negative. However, although Dice loss can calculate the similarity of two contours, it may cause the gradient to change drastically, and the training is difficult so it is not credible to a certain extent sometimes. In this experiment, we did a weighted additive combination of CE loss and Dice loss to add contour features to the classification of pixels and avoid the shock of loss in training. The new loss function is formulated as follows:

(5) $$\begin{array}{c} L_{new}=\beta \cdot L_{ce}+L_{di\;ce}\text{,} \end{array}$$

where *β* is a weight coefficient for adjusting the proportion of CE function. It is a constant in the range [0, 1], and the value of this article is 0.5. The experiment proved that this new loss function effectively improves the accuracy of crack segmentation compared to using the cross-entropy loss function alone.

## 4. Experiments

All experiments were done in the following environment: Intel (R) Xeon(R) Bronze 3204 CPU @ 1.90 GHz, 32 GB RAM, GPU Tesla V100, CentOS Linux release 7.6.1810. And experiments related to deep learning are completed under PyTorch 1.10.0. We compare the proposed method with existing representative algorithms to the performance of the model on the dataset we established and also compare the visual effects of these segmentation results.

### 4.1. Metrics

In order to evaluate our work, in addition to the visual effects of segmentation images, we introduced four authoritative evaluation indicators commonly used in semantic segmentation. All experiments are performed on the dataset we established.

Suppose *k* is the number of categories (background is excluded), *p*_*ij*_ indicates that the total number of pixels that are mispredicted. *p*_*ii*_ means, *p*_*ij*_ means *FP* and *p*_*ji*_ means *FN*. Four evaluations are

(1) PA, which means the rate of the number of predicted right pixels to total pixels. It can be expressed as follows:
  (6) $$\begin{array}{c} PA=\frac{\sum_{i=0}^{k}p_{ii}}{\sum_{i=0}^{k}\sum_{j=0}^{k}p_{ij}}. \end{array}$$
(2) MPA, which means the average pixel accuracy of each category. It can be expressed as follows:
  (7) $$\begin{array}{c} MPA=\frac{1}{k+1}\overset{k}{\underset{i=0}{\sum }}\frac{p_{ii}}{\sum_{j=0}^{k}p_{ij}}\text{.} \end{array}$$
(3) MIoU, which represents the IoU of each category. It can be expressed as follows:
  (8) $$\begin{array}{c} MIoU=\frac{1}{k+1}\overset{k}{\underset{i=0}{\sum }}\frac{p_{ii}}{\sum_{j=0}^{k}p_{ij}+\sum_{j=0}^{k}p_{ji}-p_{ii}}\text{.} \end{array}$$
(4) FWIoU, which sets the weight for IoU of each class according to the frequency of its appearance. It can be expressed as follows:
  (9) $$\begin{array}{c} FWIoU=\frac{1}{\sum_{i=0}^{k}\sum_{j=0}^{k}p_{ij}}\overset{k}{\underset{i=0}{\sum }}\frac{p_{ij}\sum_{j=0}^{k}p_{ij}}{\sum_{j=0}^{k}p_{ij}+\sum_{j=0}^{k}p_{ji}-p_{ii}}\text{.} \end{array}$$

### 4.2. Ablation Experiments

**Figure 6:**
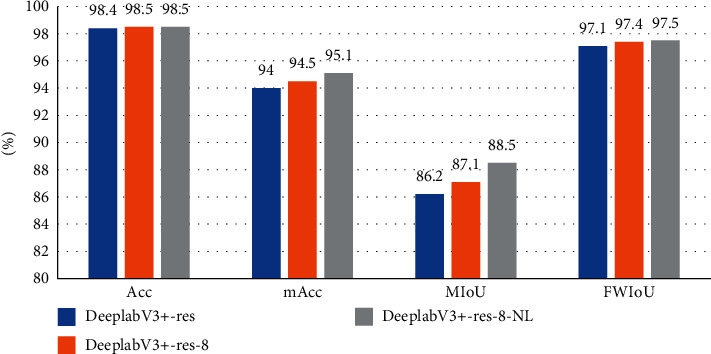
Histogram ablation experiment results with the backbone of ResNet101.

**Figure 7:**
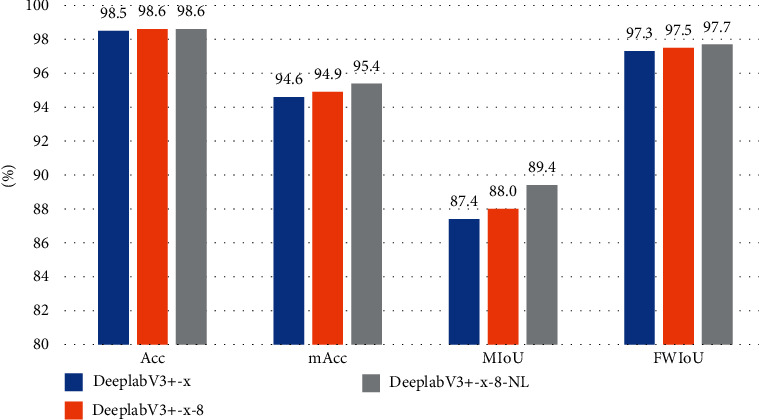
Histogram ablation experiment results with the backbone of Xception.

To scrutinize the effectiveness of the methods we proposed, we conduct experiments with two different backbones which are used in the original DeepLabV3+. Hyperparameters used by these methods are shown in Table 1. When using the ResNet101 as the backbone, the model is trained by three following strategies: (1) DeepLabV3+-res, which is an unmodified DeepLabV3+ model applying ResNet101 [40] as the backbone. (2) DeepLabV3+-res-8 changes OS to 8 and ASPP rates to (1, 12, 24, 36) on the basis of DeepLabV3+-res. (3) DeepLabV3+-x-8-NL changes loss function to the new loss on the basis of DeepLabV3+-x-8. And, the experiment results are shown in Table 2. When using the Xception as the backbone, the model is trained by three following strategies: (1) DeepLabV3+-x, which is an unmodified DeepLabV3+ model applying Xception as the backbone. (2) DeepLabV3+-x-8 changes OS to 8 and ASPP rates to (1,12,24,36) on the basis of DeepLabV3+-x. (3) DeepLabV3+-x-8-NL changes loss function to the new loss on the basis of DeepLabV3+-x-8. Results are shown in Table 3. In order to more vividly reflect the advantages of our method, histograms were drawn in Figures 6 and 7.

It can be seen in Tables 2 and 3, adjusted the OS to 8 and using new loss improve all evaluation metrics. When the backbone is RseNet101, by our methods, the Acc, mAcc, MIoU, and FWIoU improved by 0.1%, 1.1%, 2.3%, and 0.4%. When the backbone is Xception, by our methods, the Acc, mAcc, MIoU, and FWIoU improved by 0.1%, 0.8%, 2.0%, and 0.4%. Experimental results confirm the effectiveness of the proposed method.

### 4.3. Comparing with Exiting Methods

We compare the proposed method in this paper with other three typical methods: (1) FCN, the most classic semantic segmentation network. (2) U-net, the most widely used segmentation network in the medical field. (3) PSPNet, a very efficient model which applies a pyramid pooling module to fusion features on different levels. The feature extractors of all networks apply transfer learning techniques and are fine-tuned on our augmented dataset. Besides, all these models have trained 100 epochs with regular hyperparameters, and the convergence of these models was guaranteed. We also implement two different threshold segmentation methods on our test set to compare the segmentation effects between traditional methods and deep learning methods: (1) Otsu [41] (2) Max Entropy [42]. The two methods represent different automatic threshold determination methods.

**Figure 8:**
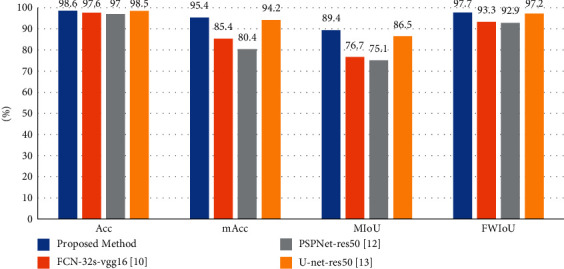
Histogram comparison of performance of our method and others.

A comparison of evaluation metrics of all these methods is shown in Table 4. As we can see, since the proportion of cracks in the images is very low, and the judgment error rate of image background pixels is low so that the total pixel accuracy of every method is not very different. However, the performance of different methods can still be judged from the remaining evaluation indicators. PSPNet and FCN may have good performance in semantic segmentation under natural conditions, but they do not perform well on the coal crack CT image dataset. U-net is designed to deal with medical images which have similarities with the images we used, so this model can have a nice performance. As the best performing comparison method, U-net achieved an Acc of 98.5%, mAcc of 94.2, MIoU of 86.5%, and a FWIoU of 97.2% which are 0.1%, 1.2%, 2.9%, and 0.5% lower than proposed method. A histogram comparison of the experimental results is shown in Figure 8.

**Figure 9:**
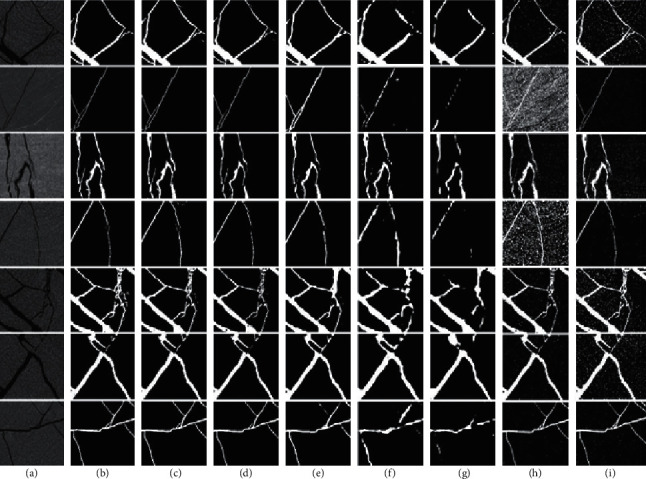
Visual effect comparison of prediction results of different methods. The columns are (a) original image, (b) ground truth, (c) our method. (d) DeepLabV3+-Xception, (e) U-net, (f) FCN32s-vgg16, (g) PSPNet, (h) Ostu, and (i) Max Entropy.

Figure 9 shows a visual effect comparison of segmentation results of all methods. It can be seen that the method we proposed has a certain improvement in the dataset in this paper. Compared with the original DeepLabV3+, the ability to capture details has been improved and cracks whose pixel values are close to the background can be identified. Many locations that should be connected become disconnected during the segmentation process of other models, this problem is also alleviated by our method. Other deep learning methods even have a large number of separation cracks sticking together, which is caused by the insufficient segmentation performance. In addition, the shape and structure of cracks is not guaranteed. Experiments show that less downsampling and the addition of the Dice loss function allow the details to be effectively preserved and recovered. Comparing deep learning methods and traditional threshold segmentation methods, the noise problem is difficult to solve for threshold even though different thresholding methods are used. Although some deep learning methods are rough for object segmentation, they often do not misidentify noise.

## 5. Conclusions

In this paper, we propose a deep learning method to complete the CT image segmentation task of coal crack CT images. Since the target in the crack image is small, and downsampling can lose the accuracy to some extent, the OS of the backbone was proposed to be reasonably adjusted to reduce the loss of accuracy and adjusted the structure of ASPP to adapt to this adjustment. In order to solve the problem of uneven sample distribution, CE loss and Dice loss were combined to define a new loss function. The experimental results show that our method is effective and has practical application value.

Nevertheless, the presented method can be improved in the following directions. First, scale-up datasets to accommodate more complex environments. And, more data will be added to this dataset which has more complex topologies and tiny targets. Moreover, we will define a new loss function according to the specific target proportion in the data set combined with probability mathematics which may be more adaptable to the needs of the field than the loss function in this paper.

## Figures and Tables

**Table 1:** Hyperparameters of six training strategies.

Batch size	LR	Epochs	LR scheduler	Weight decay	Momentum
16	0.007	100	Poly	5*e* − 4	0.9

**Table 2:** Comparison of model modifications with the backbone of ResNet101.

Methods	Acc (%)	mAcc (%)	MIoU (%)	FWIoU (%)
DeepLabV3+-res	98.4	94.0	86.2	97.1
DeepLabV3+-res-8	98.5	94.5	87.1	97.4
DeepLabV3+-res-8-NL	98.5	95.1	88.5	97.5

**Table 3:** Comparison of model modifications with the backbone of Xception.

Methods	Acc (%)	mAcc (%)	MIoU (%)	FWIoU (%)
DeepLabV3+-x	98.5	94.6	87.4	97.3
DeepLabV3+-x-8	98.6	94.9	88.0	97.5
DeepLabV3+-x-8-NL	98.6	95.4	89.4	97.7

**Table 4:** Comparison of performance of our method and others.

Methods	Acc (%)	mAcc (%)	MIoU (%)	FWIoU (%)
Proposed method	98.6	95.4	89.4	97.7
FCN-32s-vgg16 [10]	97.6	85.4	76.7	93.3
PSPNet-res50 [12]	97.0	80.4	75.1	92.9
U-net-res50 [13]	98.5	94.2	86.5	97.2

## Data Availability

The experimental data used to support the findings of this study are available from the corresponding author upon request.
